# Supplementary material for: From Virtual Reality to Regenerative Virtual Therapy: Some Insights from a Systematic Review Exploring Inner Body Perception in Anorexia and Bulimia Nervosa
Source: J Clin Med. 2022 Nov 30;11(23):7134. doi: 10.3390/jcm11237134 (PMC9737310; doi:10.3390/jcm11237134)
Supplement: Supplementary file 1 [file jcm-11-07134-s001.zip › jcm-1935526-supplementary.pdf]

Table S1

Detailed search strategy for Eating Disorders

Eating Disorders AND	PubMed	Wef of Science	PsycINFO
Internal body	769	591	1164
Body sensation	197	111	178
Proprioception	25	13	21
Interoception	118	130	98
Interoceptive	243	388	384
Vestibular	11	19	9
Autonomic system	131	101	104
Visceral	133	149	206
Internal perception	334	110	261
Body experience	682	2068	2165
**Sub total**	**2643**	**3680**	**4590**

Table S2

Detailed search strategy for Anorexia

Anorexia AND	PubMed	Wef of Science	PsycINFO
Internal body	584	387	304
Body sensation	150	88	59
Proprioception	27	12	9
Interoception	84	107	39
Interoceptive	160	306	161
Vestibular	7	16	6
Autonomic system	137	129	61
Visceral	251	331	39
Internal perception	131	70	50
Body experience	346	1061	610
**Sub total**	**1877**	**2507**	**1338**

Table S3

Detailed search strategy for Bulimia

Bulimia AND	PubMed	Wef of Science	PsycINFO
Internal body	257	180	271
Body sensation	50	39	33
Proprioception	4	5	3
Interoception	28	53	15
Interoceptive	106	220	163
Vestibular	2	6	2
Autonomic system	24	29	25
Visceral	17	48	12
Internal perception	145	35	47
Body experience	201	586	431
**Sub total**	**834**	**1201**	**1002**
